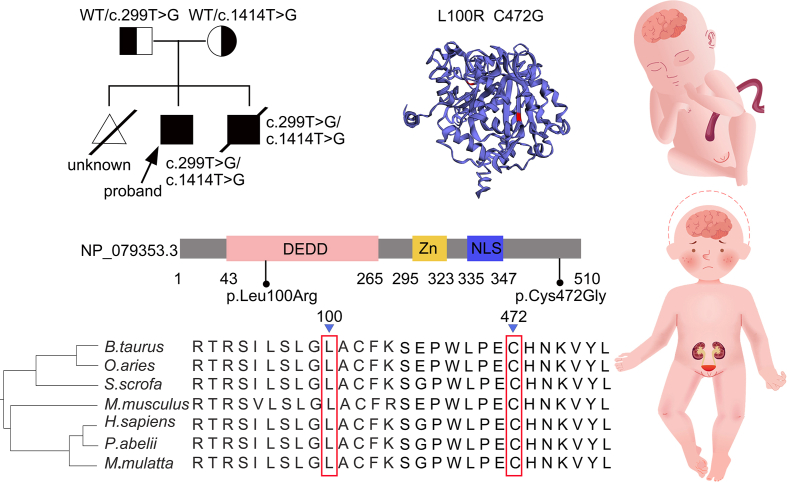# Novel heterozygous missense variants in the *TOE1* gene linked to pontocerebellar hypoplasia type 7

**DOI:** 10.1016/j.gendis.2024.101290

**Published:** 2024-04-08

**Authors:** Aijun Yang, Xiaoli Kong, Qin Wang, Runqing Miao, Haixiang Ma, Anzhuo Chu, Zhengtong Wang, Jiaqing Lu, Bo Liu, Bingcheng Mu, Runhan Guo, Jiayi Li, Xiaoxiao Gongye, Huabao Xiong, Tao Zhong

**Affiliations:** a Jining Medical University, Jining, Shandong 272067, China; b The University of Manchester, Oxford Rd, Manchester M13 9PL, UK

Pontocerebellar hypoplasia type 7 (PCH7) (OMIM #614969) stands as a rare and severe neurodegenerative syndrome marked by distinct characteristics, including neurological decline, hypoplasia in the pons and cerebellum, muscle hypotonia, irregular breathing, and hypogonadism.^1^ Furthermore, individuals with 46, XY karyotypes display feminine genitalia, whereas those with 46, XX karyotypes exhibit atrophic ovaries and lack menarche in PCH7 patients.^2^ Recent investigations have pinpointed variants in the EGR1 protein 1 gene (*TOE1*) as the genetic culprits behind PCH7 onset.^3^ *TOE1* primarily situated within the Cajal bodies of the nucleus has been identified. In this cellular compartment, *TOE1* functions as a 3-exonuclease, aiding in the maturation of small nuclear RNAs (snRNAs) and the processing of snRNA 3′-tails.^4^ Disruptions in snRNA processing may contribute to severe neurodegenerative disorders.

In March 2020, the 37th-week gestation examination during a woman's first pregnancy indicated a small fetal cerebellum, slender corpus callosum, and enlarged ventricles in our hospital. The pregnancy was terminated, but genetic testing was not performed on the induced fetus. On November 20, 2021, a prenatal ultrasound scan at 29 weeks of the mother's second pregnancy revealed bilateral ventricle enlargement as shown in Figure 1H. Concurrently, a prenatal MRI scan indicated significant bilateral lateral ventricle enlargement, reduced size of the bilateral cerebellar hemispheres, notable cisterna occipitalis enlargement, and thinning of the corpus callosum as shown in Figure 1A and B. Whole-exome sequencing results identified biallelic variants in *TOE1* for this fetus as shown in Fig. 1N, including two novel heterozygous missense variants *TOE1*:c.1414T > G;p.(Cys472Gly) and *TOE1*:c.299T > G;p.(Leu100Arg). Despite being informed of the risk of fetal abnormalities, the mother did not terminate the pregnancy. The proband, delivered vaginally at 42 gestational weeks, had a birth weight of 3650 g and a body length of 50 cm. The child exhibited female-like external genitalia without a vaginal orifice. Raised as a female, the child's head circumference measured 36 cm one month after birth. Subsequent MRI revealed a small cerebellar volume, a slender and partially absent corpus callosum, widening of the cisterna magna, narrowing of the aqueduct of sylvius, dilated supratentorial ventricles, and pachygyria as shown in Figure 1C–F. Ovaries and uterus were not visible by type-B ultrasonic detection at 5 months after birth as shown in Figure 1I. Chromosome karyotyping revealed 46, XY. At 1 year and 10 months, the proband weighed 6000 g, measured 73 cm in body length, and had a head circumference of 41 cm. The child exhibited developmental retardation, loss of facial expression, high muscle tone, constant plantarflexion of both feet, inability to stand and chew, lack of eye movement following object motion, persistent tracheal wheezing rale, and a diet comprising fluid food. Genital examination revealed female-like external genitalia with no observed testicles. Moreover, the vaginal orifice of the proband was atresia, and a small penis was located in the perineum, resembling the external orifice of the urethra, from which urine was excreted as shown in Figure 1J.

**Figure 1:**
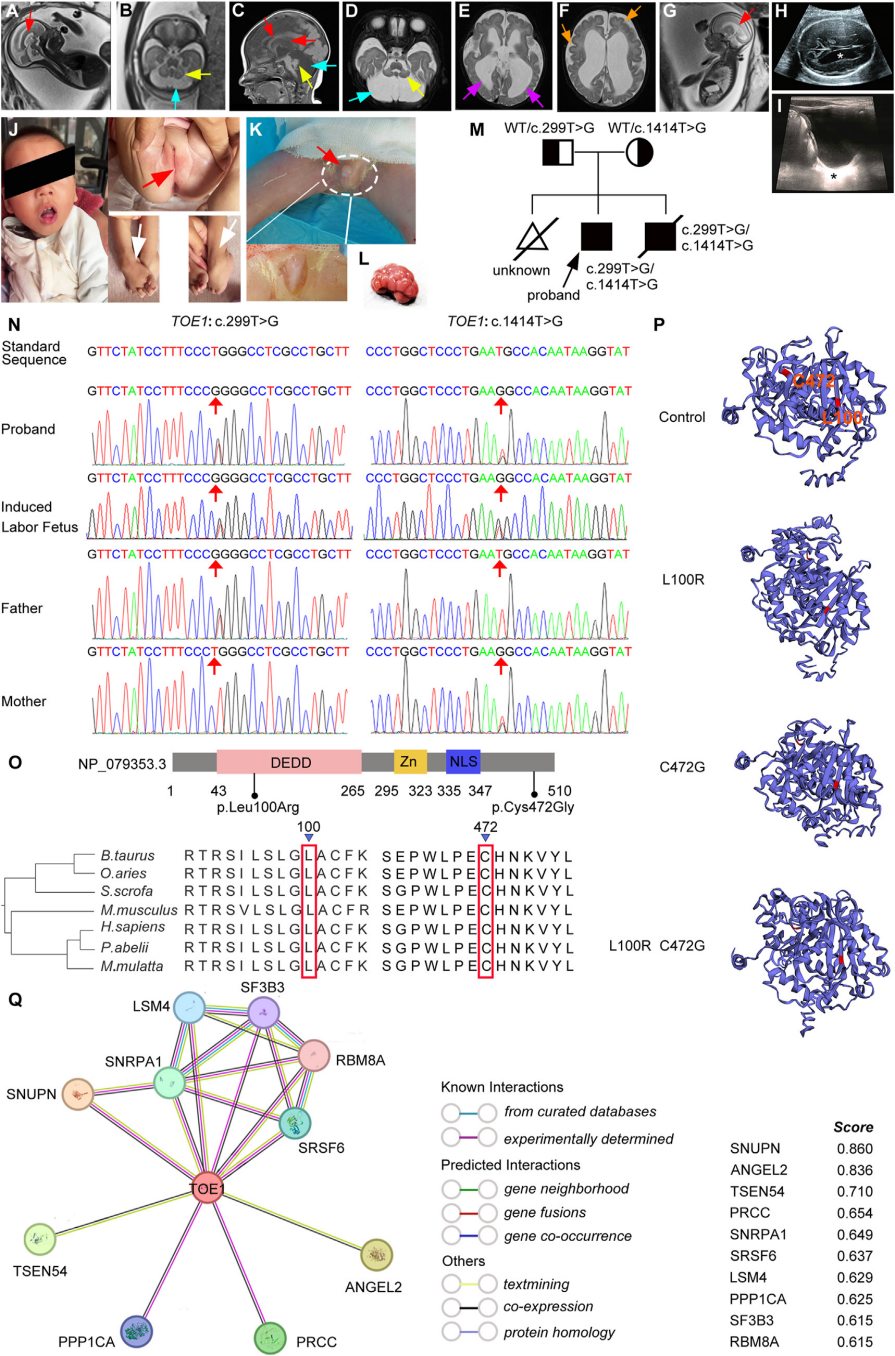
Clinical features and sequence analysis of *TOE1* Variant patients. **(A)** A prenatal MRI scan at 29 weeks of the second pregnancy showed a thinning corpus callosum. **(B)** MRI in the same batch as Figure 1A showed reduced cerebellar volume (yellow arrow) and enlarged cerebellomedullary cistern (blue arrow). **(C)** Type-B ultrasound examination at 29 weeks of the second pregnancy showed enlarged lateral ventricles (white asterisk). **(D)** MRI in the same batch as Figure 1C showed reduced cerebellar volume (yellow arrow) and enlarged cerebellomedullary cistern (blue arrow). **(E)** MRI in the same batch as Figure 1C showed an enlarged supratentorial ventricle (violet arrow). **(F)** MRI in the same batch as Figure 1C showed pachygyria (orange arrow). **(G)** A prenatal MRI scan at 25 weeks of the third pregnancy showed a thinning corpus callosum (red arrow). **(H)** MRI of the fetus one month after birth showed a thinning and partially absent corpus callosum (red arrow), enlarged cerebellomedullary cistern (blue arrow), and reduced cerebellar volume (yellow arrow). **(I)** Type-B ultrasound examination of the fetus one month after birth showed the absence of ovaries and uterus (black asterisk). **(J)** The proband is stunted, has a loss of facial expression, and has the appearance of female-like external genitalia without the vaginal orifice (red arrow). Both feet of the child patient are constantly in plantarflexion, resulting in an inability to stand (white arrow). **(K)** Short penis and missing testicles in the proband. **(L)** Polycystic changes of the fetus's kidney of *TOE1* variants. **(M, N)** The same genetic variant occurred in both the proband and the induced labor fetus, *i.e.*, a maternally inherited missense variant c.1414T > G and a paternally inherited missense variant c.299T > G in *TOE1*. Parents are clinically phenotypically normal. **(O)** Variants p.Leu100Arg and p.Cys472Gly of *TOE1* were conservative during the evolution of mammals. **(P)** Alterations in amino acids of p.Leu100Arg and p.Cys472Gly could result in structural changes in the *TOE1* protein. **(Q)** Summarized and predicted interaction proteins of *TOE1* by String online service.

To ensure a healthy pregnancy, the patient conceived for the third time and presented at our hospital at 25 weeks of gestation. However, an MRI scan revealed a small cerebellar volume and slender corpus callosum again as shown in Figure 1G. Whole-exome sequencing data of the fetus indicated the recurrence of the same variant in *TOE1* as in the previous pregnancies as shown in Figure 1N. The mother opted to terminate the pregnancy, allowing us to study induced fetal tissue and organs to elucidate the pathological causes. On October 16, 2023, the mother delivered a stillborn child transvaginally, revealing a short penis and pathological anatomy indicating the absence of testes. Karyotyping confirmed 46, XY. We generate a pedigree tree to illustrate the presence of these variants among the parents and the patients as shown in Figure 1M. Additionally, polycystic changes were observed in the fetus's kidneys as shown in Figure 1L.

Subsequently, the novel heterozygous variants in the *TOE1* gene were validated using standard Sanger sequencing. The *TOE1*:c.299T > G;p.(Leu100Arg) variant, causing an amino acid change of p.Leu100Arg in exon 4, was inherited from the father. Conversely, the *TOE1*:c.1414T > G;p.(Cys472Gly) variant, resulting in an amino acid change of p.Cys472Gly in exon 8, was inherited from the mother. Importantly, these two sites were highly conserved in phylogeny as shown in Figure 1O. ITASSER predicted both variants to induce structural changes as shown in Figure 1P. Neither variant had been documented in public databases like OMIM, HPO, CHPO, or Orphanet. Summarization and prediction of interaction proteins were performed using the String online service, identifying several protein molecules capable of interacting with *TOE1* as shown in Figure 1Q.

TOE1 comprises 510 amino acids, encompassing a crucial Asp-Glu-Asp-Asp (DEDD) deadenylase domain, a C3H-type zinc finger, and a nuclear localization signal. The DEDD domain is essential for *TOE1*'s exonuclease activity, as demonstrated by the inability of a deadenylase-dead mutant to restore telomerase activity in *TOE1*-deficient cells. The removal of the nuclear localization signal disrupts *TOE1*'s localization in Cajal bodies, affecting its ability to bind to DKC1 and telomerase.^5^ We have summarized and compared the phenotypic characteristics and variant sites of our patients with those previously documented cases as shown in Table S1. The novel variant *TOE1*:c.299T > G;p.(Leu100Arg) in our patient, situated in the DEDD domain, exhibited a severe phenotype compared with patients with other variants in the same region. Additionally, the stillborn fetus from the mother's third pregnancy exhibited specific polycystic changes in the kidneys, a novel observation. The surviving child continues to experience tracheal wheezing rales and presents as gender-reversed with 46, XY chromosomes, explaining the female-like external genitalia without a vaginal orifice, and the inability to detect ovaries and uterus by type-B ultrasound. This could be attributed to the simultaneous presence of the *TOE1*:c.1414T > G;p.(Cys472Gly) variant, partially exacerbating the variation in the structure and function of *TOE1*.

In conclusion, we confirmed novel variants in our patients. Until then, no evidence proves a causal association of *TOE1*:c.1414T > G;p.(Cys472Gly) and *TOE1*:c.299T > G;p.(Leu100Arg) variants with PCH7. Furthermore, future systematic *in vitro* experiments exploring the impact of these missense variants and verifying their interactions with *TOE1* protein function can provide additional insights into the observed phenotypic distinctions among patients. Our findings not only provide additional evidence of the correlation between *TOE1* variants and neurological syndromes but also highlight the capability of these variants to manifest in diverse clinical presentations. These results enhance comprehension of the phenotypes linked to *TOE1* variants. In addition, these findings expand the spectrum of genetic and phenotypic manifestations observed in PCH7 disorders. Our discoveries offer helpful insights for distinguishing rare neurodevelopmental disorders and provide a reference for genetic counseling services. Couples who are carriers of *TOE1* variants may consider *in vitro* fertilization combined with preimplantation genetic testing as a preventive measure to avoid the birth of offspring with PCH7.

## Ethics declaration

We are highly obliged to the family members for their cooperation and participation in this study. The study protocol obtained approval from the Ethics Committee of the affiliated hospital of Jining Medical University (2023-11-C020). Written informed consent for the presentation and publication of this study was obtained from the legal guardians of the patients. The use of the child's photo was also approved by the legal guardians of the patient.

## Author contributions

T.Z. and H.B.X. conceived the study. A.J.Y., X.L.K., and T.Z. designed the experiments. X.L.K., Q.W., R.Q.M., and H.X.M. conducted the experiments. A.Z.C., Z.T.W., J.Q.L., and B.L. performed the data analyses. B.C.M., R.H.G., J.Y.L., and X.X.G.Y. participated in collecting clinical image data. T.Z. and A.J.Y. wrote and edited the original draft. All authors approved its submission to Genes & Diseases.

## Conflict of interests

The authors declared no competing interests.

## Funding

This study was supported by grants from the 10.13039/100014717National Natural Science Foundation of China (No. 82171810) and the Program of Shandong Provincial TCM Sci-Tech Project (M-2023210).

## Figures and Tables

**figs1:**