# Supplementary material for: Novel heterozygous missense variants in the TOE1 gene linked to pontocerebellar hypoplasia type 7
Source: Genes Dis. 2024 Apr 8;12(1):101290. doi: 10.1016/j.gendis.2024.101290 (PMC11549980; doi:10.1016/j.gendis.2024.101290)
Supplement: Multimedia component 1 [file mmc1.docx]

**MATERIAL AND METHODS**

**Patients**

The study protocol obtained approval from the Ethics Committee of the affiliated hospital of Jining Medical University (2023-11-C020). Written consent was acquired from the legal guardians of the patient. The proband, a 1-year and 10-month-old child, is the second offspring of the mother. The first pregnancy was terminated due to the potential for fetal brain dysplasia, as indicated by prenatal examination. In April 2023, the woman was pregnant for the third time. On October 11, 2023, prenatal MRI revealed a small cerebellum volume and slender corpus callosum at 25 weeks of the third fetus. These clinical features indicated fetal brain deficiency, causing confusion and a desire for a healthy offspring. No family history of genetic diseases was reported, and prenatal fetal MRI results and ultrasound scan data were available.

**Gene tests and genetic analysis**

WES and variant analysis were conducted following established guidelines^1^. Two WES and analyses were performed before and after. Amniotic fluid samples (10ml) were collected during the second and third pregnancies, and parental blood (10ml each) was obtained each time. Genomic DNA extraction used the High Sensitivity DNA kit (Geneon BioTech, Changchun, China). Exon enrichment and amplification were carried out using the WES v1.0 kit (Basecare, Suzhou, China). High-throughput sequencing was performed on the MGISEQ-2000 (BGI, Shenzhen, China) platform. Raw data underwent filtering, variant annotation, and interpretation using the GeneX system (Basecare, Suzhou, China). Pathogenicity assessment of variants followed the American College of Medical Genetics and Genomics (ACMG) guidelines.

**Prediction of variants effects on *TOE1* structure and interaction proteins**

Amino acid sequence comparison and phylogenetic tree construction of *TOE1* were conducted using FigTree software (V1.4.3). The three-dimensional structure of the *TOE1*-encoded protein was predicted using the ITASSER online server, employing a threading algorithm. The protein structure was visualized with SwissPdb Viewer software (v.4.1.0). Additionally, predicted interaction proteins of *TOE1* were summarized and disclosed using the String online service.

**REFERENCES**

1. Richards S, Aziz N, Bale S *et al*: Standards and guidelines for the interpretation of sequence variants: a joint consensus recommendation of the American College of Medical Genetics and Genomics and the Association for Molecular Pathology. *Genet Med* 2015; **17:** 405-424.
