# Supplementary material for: Novel heterozygous missense variants in the TOE1 gene linked to pontocerebellar hypoplasia type 7
Source: Genes Dis. 2024 Apr 8;12(1):101290. doi: 10.1016/j.gendis.2024.101290 (PMC11549980; doi:10.1016/j.gendis.2024.101290)
Supplement: Multimedia component 2 [file mmc2.docx]

**Table S1: Phenotypic and EGR1 Protein 1 Gene (*TOE1*) Variants Comparison of Our Patients with Reported Patients**

| References | This Study | PMID: 28092684 | PMID: 37608778 | PMID: 37635087 | PMID: 34716526 | PMID: 36738896 | PMID: 36076253 |
| --- | --- | --- | --- | --- | --- | --- | --- |
| EGR1 Protein 1 Gene (*TOE1*)  NM_025077.4 | c.299T>G  and c.1414T>G | c.658G>A  c.443T>A  c.307G>A  c.195+5G>C  c.219G>C and c.693T>A  c.757C>T and c.957C>T  c.518T>G and c.938_939DdelCA  c.518T>G and c.957C>A  c.716T>C and c.1487C>T | c. 553C> T  and  c. 562G> T | c.716T > C and c.955C > T | c.237-2A > G and  c.551G > T | c.911C>T and c.161C>T | c.572A > G |
| No. of Patients | 2 | 12 | 2 | 2 | 1 | 1 | 2 |
| Microcephaly | + | NA | 1/2 | + | - | - | 1/2 |
| Small Cerebellar Volume | + | + | + | + | - | + | + |
| Ventriculomegaly | + | + | + | + | + | + | NA |
| Thin Corpus Callosum | + | + | + | 1/2 | - | + | + |
| Abnormal Lateral Fissure | + | NA | - | 1/2 | - | + | + |
| Underdeveloped Insula | - | na | - | 1/2 | - | - | na |
| Pons And Brainstem Hypoplasia | - | + | + | + | - | + | - |
| Psychomotor Delay | + | na | + | na | + | + | na |
| Hypotonia | - | NA | NA | NA | +  (upper limbs) | + | NA |
| Hypertonia | + | NA | + | NA | +  (lower limbs) | - | NA |
| Breathing Abnormalities | + | NA | - | NA | - | - | NA |
| Hypogonadism | + | + | 1/2 | NA | - | + | 1/2 |
| Polycystic Kidney | + | NA | NA | NA | NA | NA | NA |
