# Supplementary material for: Novel heterozygous missense variants in the TOE1 gene linked to pontocerebellar hypoplasia type 7
Source: Genes Dis. 2024 Apr 8;12(1):101290. doi: 10.1016/j.gendis.2024.101290 (PMC11549980; doi:10.1016/j.gendis.2024.101290)
Supplement: Multimedia component 3 [file mmc3.docx]

**Novel compound heterozygous missense variants in the TOE1 gene linked to** **pontocerebellar hypoplasia type 7**

**
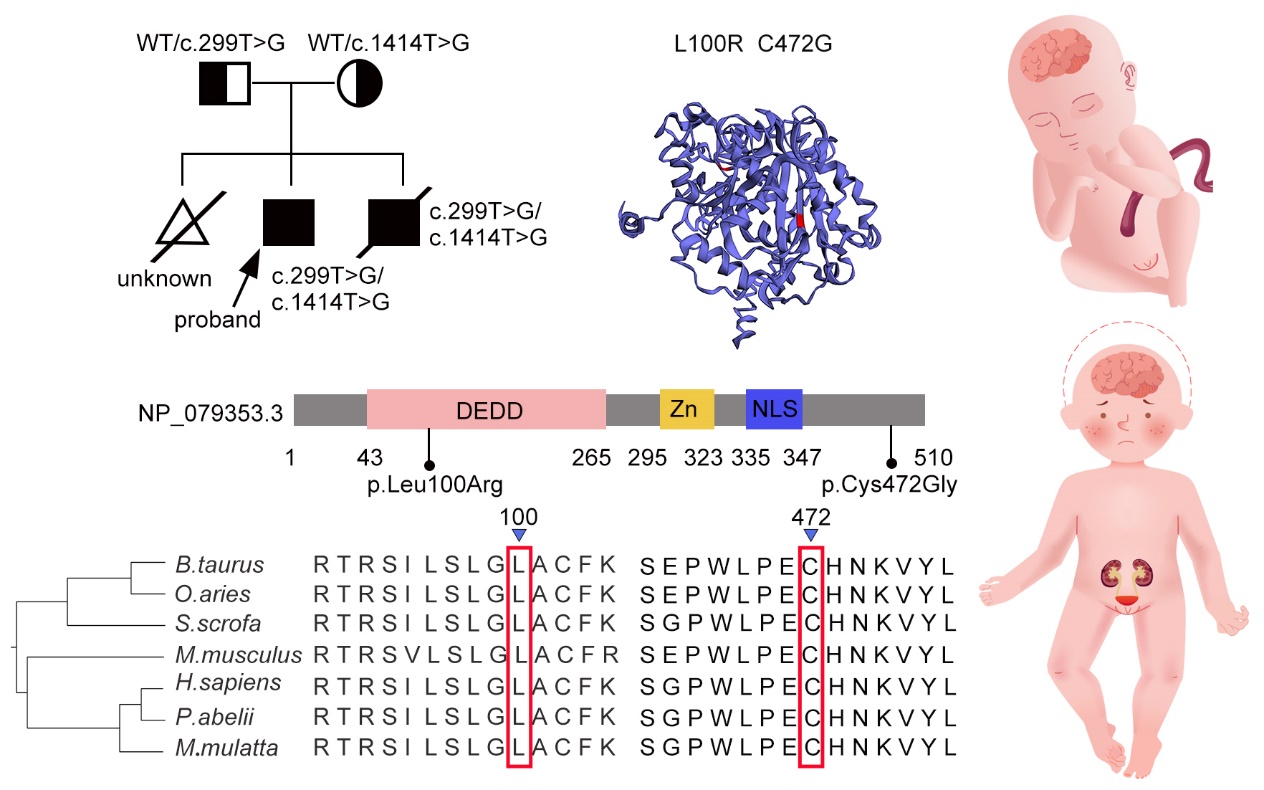
**

Our research identified two previously unreported compound heterozygous variants of *TOE1* associated to pontocerebellar hypoplasia type 7, including a maternal inherited missense variant c.1414T>G (p.Cys472Gly) and a paternal missense variant c.299T>G (p.Leu100Arg). The two sites were highly conservative in phylogeny, variants will change TOE1 protein structure. Clinical features of the fetus were present brain deformities (pachygyria, thinning corpus callosum and reduced cerebellar volume) in pregnancy. Patients exhibited developmental retardation, loss of facial expression, high muscle tone, constant plantarflexion of both feet, inability to stand and chew, lack of eye movement following object motion, persistent tracheal wheezing rale, after birth. Moreover, we found polycystic morphology of the kidney in one of offspring patients.

**Summary**

Pontocerebellar hypoplasia type 7 (PCH7) (OMIM # 614969) is a rare and severe neurodegenerative syndrome. Distinctive characteristics of PCH7 include neurological decline, along with hypoplasia in the pons and cerebellum, muscle hypotonia, irregularities in breathing, and hypogonadism[1-3]. Moreover, based on the previous study, individuals with 46, XY karyotypes exhibit feminine genitalia, while those with 46, XX karyotypes present with atrophic ovaries and absence of menarche in PCH7 patients[4-6]. Therefore, investigating the pathogenic mechanism of PCH7 and preventing its occurrence in offspring is worth doing.

Recently, studies discovered that bi-allelic variants in the EGR1 protein 1 (*TOE1*) gene were identified as the genetic variants responsible for the onset of PCH7[7, 8]. The *TOE1* gene is situated on chromosome 1p34.1 and follows an autosomal recessive inheritance pattern[9]. In previous studies, *TOE1* is primarily localized within the Cajal bodies of the nucleus[10-12]. Within this specific cellular compartment, *TOE1* functions as a 3-exonuclease, facilitating the maturation process of small nuclear RNAs (snRNAs) and the processing of snRNA 3'-tails[7, 11, 13]. Dysfunctions in snRNA processing may serve as a shared factor contributing to severe neurodegenerative disorders[14-16].

In this study, we verified a novel *TOE1* bi-allelic variant in our patients with cerebellum dysplasia, microcephaly, terrible intellectual disability, developmental delay, dystonia, and sex reversal. WES analysis uncovered two previously unreported compound heterozygous variants of *TOE1* in our patients, including a maternal inherited missense variant c.1414T>G (p.Cys472Gly) and a paternal missense variant c.299T>G (p.Leu100Arg). Clinical features of the children patient were mostly concordant with previous reports but brain deformities (pachygyria, thinning corpus callosum and reduced cerebellar volume) are more pronounced. Moreover, we found polycystic morphology of the kidney in one of the fetuses. Associated interaction proteins and TOE1 altering structure about variants were predicted by some bioinformatics analysis. The discovery broadens the range of genotypic and phenotypic manifestations linked to *TOE1* gene variants, contributing to a deeper comprehension of the disease.

The reproductive quality has been improved dramatically by advances in prenatal diagnosis strategies. Nevertheless, there exists a large quantity of congenital malformation attributed to the uncertain gene variants in nature. To investigate the major causes of congenital malformation and prevent diseases occurrence in offspring is worth doing. Herein, we describe a rare case of an asymptomatic Chinese couple generating their offspring with cerebellum dysplasia, microcephaly, terrible intellectual disability, developmental delay, and dystonia. Using WES technology, two previously unreported compound heterozygous variants of *TOE1* in their children were successfully identified, including a maternal inherited missense variant c.1414T>G (p.Cys472Gly) and a paternal missense variant c.299T>G (p.Leu100Arg). In previous studies, multiple variants in *TOE1* were associated with PCH7. We have summarized and contrasted the phenotypic characteristics of our patients with those of 21 patients previously documented in the literatures[7-9, 17-20]. As shown in our research, the clinical phenotypes of two offspring patients in this study were generally consistent with those previously reported in the literature. The reduced cerebellum volume, thinning corpus callosum and gonadal malformation are more obvious. In addition, the induced labor fetus of the mother’s third pregnancy showed specific polycystic change in kidneys, which has not been reported before. The surviving child persists with tracheal wheezing rale and presents as gender reversed with 46, XY chromosomes. This can explain why this child has the appearance of female-like external genitalia without vaginal orifice, and why ovaries and uterus couldn’t be detected by type-B ultrasound.

TOE1 consists of 510 amino acids and encompasses a Asp-Glu-Asp-Asp (DEDD) deadenylase domain, a C3H-type zinc finger (ZN), and a nuclear localization signal (NLS)[21]. Previous studies have been disclosed some variants of TOE1 as table2. The DEDD domain is crucial for the exonuclease activity of *TOE1*[7]. This is evident from the inability of a deadenylase-dead mutant to restore telomerase activity in *TOE1*-deficient cells. Elimination of the NLS impedes the localization of TOE1 in Cajal bodies, disrupting its ability to bind DKC1 and telomerase[21]. Novel variants of c.299T>G (p.Leu100Arg) in our patient being situated in the DEDD domain, their phenotype exhibited severe characteristics in comparison to patients harboring other variants in the same region. This could be attributed to the simultaneous presence of the c.1414T>G (p.Cys472Gly) variant, which partially aggravates the variation of structure and function of TOE1. Besides, interaction proteins were predicted by some biology software or online service. In the future, systematic in vitro experiments exploring the impact of these missense variants and verifying its interactions with TOE1 protein function can offer further insights into the phenotypic distinctions observed among patients.

In summary, we verified a novel bi-allelic variant in our patient’s offspring with cerebellum dysplasia, microcephaly, terrible intellectual disability, developmental delay, dystonia, and sex reversal. To further achieve a better comprehension of the phenotype linked to *TOE1* variation, it is essential to thoroughly evaluate and test PCH7 patients. Our findings not only provide additional evidence regarding the association between TOE1 variants and neurological syndromes but also demonstrate the ability of these variants to result in diverse clinical manifestations. These results have broadened the spectrum of genetic and phenotypic manifestations observed in PCH7 disorders. Our discoveries provide valuable insights for distinguishing rare neurodevelopmental disorders and are instrumental in offering genetic counseling services. Couples who are carriers of TOE1 variants can consider the option of in vitro fertilization combined with preimplantation genetic testing as a means to prevent the birth of offspring with PCH7.

**References**

1. van Dijk, T., et al., *What's new in pontocerebellar hypoplasia? An update on genes and subtypes.* Orphanet J Rare Dis, 2018. **13**(1): p. 92.

2. Rusch, C.T., et al., *Pontocerebellar Hypoplasia: a Pattern Recognition Approach.* Cerebellum, 2020. **19**(4): p. 569-582.

3. Anderson, C., et al., *Early pontocerebellar hypoplasia with vanishing testes: A new syndrome?* Am J Med Genet A, 2011. **155A**(4): p. 667-72.

4. Rudnik-Schoneborn, S., P.G. Barth, and K. Zerres, *Pontocerebellar hypoplasia.* Am J Med Genet C Semin Med Genet, 2014. **166C**(2): p. 173-83.

5. Mahbubul Huq, A.H. and M.A. Nigro, *XY sex reversal and a nonprogressive neurologic disorder: a new syndrome?* Pediatr Neurol, 2000. **23**(4): p. 357-60.

6. Siriwardena, K., et al., *XY sex reversal, pontocerebellar hypoplasia and intellectual disability: confirmation of a new syndrome.* Am J Med Genet A, 2013. **161A**(7): p. 1714-7.

7. Lardelli, R.M., et al., *Biallelic variants in the 3' exonuclease TOE1 cause pontocerebellar hypoplasia and uncover a role in snRNA processing.* Nat Genet, 2017. **49**(3): p. 457-464.

8. Nuovo, S., et al., *Refining the variantal spectrum and gene-phenotype correlates in pontocerebellar hypoplasia: results of a multicentric study.* J Med Genet, 2022. **59**(4): p. 399-409.

9. Wu, Z.F., et al., *Clinical and genetic characterization of a Chinese family with pontocerebellar hypoplasia type 7.* Am J Med Genet A, 2023.

10. Fong, K.W., et al., *Whole-genome screening identifies proteins localized to distinct nuclear bodies.* J Cell Biol, 2013. **203**(1): p. 149-64.

11. Son, A., J.E. Park, and V.N. Kim, *PARN and TOE1 Constitute a 3' End Maturation Module for Nuclear Non-coding RNAs.* Cell Rep, 2018. **23**(3): p. 888-898.

12. Wagner, E., S.L. Clement, and J. Lykke-Andersen, *An unconventional human Ccr4-Caf1 deadenylase complex in nuclear cajal bodies.* Mol Cell Biol, 2007. **27**(5): p. 1686-95.

13. Lardelli, R.M. and J. Lykke-Andersen, *Competition between maturation and degradation drives human snRNA 3' end quality control.* Genes Dev, 2020. **34**(13-14): p. 989-1001.

14. Jia, Y., J.C. Mu, and S.L. Ackerman, *Variant of a U2 snRNA gene causes global disruption of alternative splicing and neurodegeneration.* Cell, 2012. **148**(1-2): p. 296-308.

15. Budde, B.S., et al., *tRNA splicing endonuclease variants cause pontocerebellar hypoplasia.* Nat Genet, 2008. **40**(9): p. 1113-8.

16. Tian, H., Z. Hu, and C. Wang, *The Therapeutic Potential of tRNA-derived Small RNAs in Neurodegenerative Disorders.* Aging Dis, 2022. **13**(2): p. 389-401.

17. Guo, H., et al., *Genetic and prenatal diagnosis of a Chinese pedigree with pathogenic TOE1 variants causing pontocerebellar hypoplasia type 7.* J Matern Fetal Neonatal Med, 2023. **36**(2): p. 2250895.

18. Chen, H., et al., *Novel compound heterozygous variant of TOE1 results in a mild type of pontocerebellar hypoplasia type 7: an expansion of the clinical phenotype.* Neurogenetics, 2022. **23**(1): p. 11-17.

19. Wang, C., et al., *Novel compound heterozygous missense variants in TOE1 gene associated with pontocerebellar hypoplasia type 7.* Gene, 2023. **862**: p. 147250.

20. Bilge, S., et al., *Clinical, radiological, and genetic variation in pontocerebellar hypoplasia disorder and our clinical experience.* Ital J Pediatr, 2022. **48**(1): p. 169.

21. Deng, T., et al., *TOE1 acts as a 3' exonuclease for telomerase RNA and regulates telomere maintenance.* Nucleic Acids Res, 2019. **47**(1): p. 391-405.
